# Ultrasound-Assisted Preparation of Chitosan Oligosaccharide-Stabilized Thyme Oil-in-Water Nanoemulsions: Enhanced Storage Stability and Antimicrobial Properties

**DOI:** 10.3390/foods14172930

**Published:** 2025-08-22

**Authors:** Hao Wang, Qirong Dong, Wenyue Wang, Jin Chen, Wenjun Wang, Zhongxiang Fang, Runan Zhao

**Affiliations:** 1 College of Biosystems Engineering and Food Science, National-Local Joint Engineering Research Center of Intelligent Food Technology and Equipment, Zhejiang Key Laboratory of Agro-Food Resources and High-Value Utilization, Fuli Institute of Food Science, Zhejiang University, Hangzhou 310058, China; vinnwh@163.com (H.W.); deerqr@163.com (Q.D.); cjdlgydx@163.com (J.C.); wangwj@zju.edu.cn (W.W.); 2 School of Agriculture, Food and Ecosystem Sciences, Faculty of Science, The University of Melbourne, Parkville 3010, Australia; 15238646131@163.com

**Keywords:** thymol, chitosan emulsion, co-emulsifier interface layer, stability, antimicrobial activity

## Abstract

Thyme oil (TO), an aromatic compound derived from *Thymus species*, exhibits potent antioxidant and antibacterial properties. To address its defects of high volatility and susceptibility to oxidation, TO was encapsulated in chitosan oligosaccharide (COS)-stabilized oil-in-water emulsions using a two-step emulsification method with ultrasound assistance. The droplet size of TO-in-water emulsions decreased significantly with increasing ultrasound power and treatment time, achieving sizes below 240 nm with an encapsulation efficiency exceeding 90%. The COS interface layer, combined with polyvinyl alcohol (PVA), effectively enhanced emulsion stability by preventing phase separation and maintaining droplet size and zeta potential during storage. Compared to its free form, the encapsulation of TO in the emulsion significantly improved the antioxidant activities, as evidenced by the enhanced ABTS (1.25-fold) and DPPH (1.33-fold) radical scavenging activities, at equivalent concentrations. Additionally, the TO emulsions exhibited superior antibacterial and antifungal properties, with minimum inhibitory concentration (MIC) values reduced by half and effective inhibition of *Escherichia coli*, *Staphylococcus aureus*, and *Penicillium italicum* growth. These findings highlight the potential of TO emulsions as an effective delivery system for improving the functionality and stability of TO in fresh food preservation applications.

## 1. Introduction

Thyme oil (TO), derived from *Thymus* species, is renowned for its potent antioxidant and antimicrobial properties, attributed to its bioactive compounds, such as thymol and carvacrol [1,2]. Despite its potential, the practical application of TO is hindered by its high volatility, hydrophobicity, and susceptibility to oxidation [3,4]. Encapsulation of TO has been widely reported to improve its functional performance compared to free oils, including enhanced bioavailability, sustained release, and better protection of active compounds during storage, ultimately leading to improved antimicrobial and antioxidant efficacy in practical applications [5,6,7]. Oil-in-water (O/W) emulsions are widely recognized as effective systems for encapsulating, protecting, and delivering lipophilic active ingredients, such as essential oils (EO) [7,8]. These systems improve the hydrophilicity and bacteriostatic activity of EO, enhancing their functionality in various applications [2,3]. However, one major challenge associated with EO-loaded emulsions is the phenomenon of Ostwald ripening, which compromises emulsion stability [9]. This instability arises from the higher solubility and permeability of smaller oil droplets, which merge into larger droplets, leading to phase separation and precipitation [10]. Conventional strategies to mitigate Ostwald ripening involve using water-insoluble triglycerides as ripening inhibitors. However, these inhibitors can diminish the antimicrobial potency of EO, necessitating alternative approaches to stabilize emulsions without compromising their functional properties [11]. Hence, we aimed to explore an alternative approach to prevent Ostwald ripening of EO without ripening inhibitors.

Chitosan oligosaccharides (COS) are low-molecular-weight derivatives of chitosan obtained through enzymatic or chemical hydrolysis of natural sources, like shrimp and crab shells [12]. COS has garnered significant attention due to its enhanced solubility and bioactivity compared to its parent polymer [13]. It exhibits a wide range of beneficial properties, including antimicrobial, antioxidant, and immunomodulatory activities, making it suitable for applications in pharmaceutical, agriculture, and food industries [14]. Additionally, COS retains the biocompatibility of chitosan but offers improved activity, allowing for versatile use in stabilizing emulsion systems. According to our previous works, chitosan can react with cinnamaldehyde (CA) at the oil–water interface to form a solid-like interface layer through the Schiff-base reaction to effectively inhibit Ostwald ripening and stabilize pure essential oil emulsions [11,15]. Given that COS has the same amino group as chitosan, COS is also expected to form a similar Schiff-base reaction with CA, resulting in a solid-like interfacial layer that could further stabilize oil droplets and inhibit TO diffusion, and the solid-like interfacial layer would stabilize oil droplets, potentially inhibiting the diffusion of TO molecules, thereby improving emulsion stability.

**Scheme 1:**
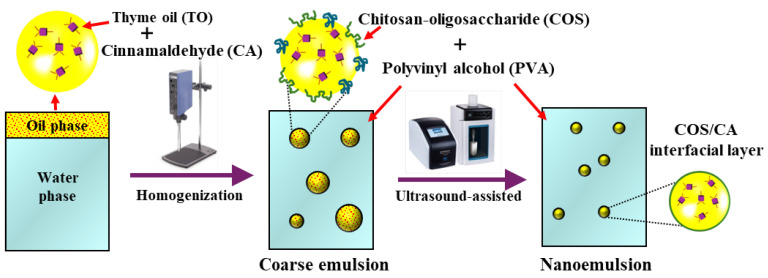
Schematic illustration of COS-stabilized thyme oil-in-water emulsion preparation by an ultrasonic-assisted method.

This study used the interfacial layer formed by COS and CA to stabilize TO emulsions (Scheme 1). Meanwhile, polyvinyl alcohol (PVA) was incorporated as a thickener to enhance the viscosity of the continuous phase and reduce gravitational separation. It was also used as a co-emulsifier to help stabilize the oil–water interface [11]. Additionally, ultrasound-assisted emulsification was used to achieve smaller droplet sizes and improve emulsion homogeneity, enhancing stability and functionality [7,16]. This approach is innovative in combining COS/CA interfacial chemistry with PVA co-emulsification, offering dual stabilization and improved functional performance, which has not been extensively reported. This study systematically investigated the effects of ultrasonic power, processing time, and TO concentration on emulsion droplet size and storage stability. Furthermore, the antioxidant and antimicrobial activities of encapsulated TO were evaluated and compared to those of free TO. Beyond its demonstrated efficacy in enhancing the stability and bioactivity of TO, the developed emulsion system holds promise for broader applications, including active food packaging, surface coatings for fresh produce, and potential use as a delivery vehicle for lipophilic bioactives in pharmaceutical formulations. By integrating these findings, the research aimed to provide a foundation for applying TO emulsions in food preservation and other relevant fields.

## 2. Materials and Methods

### 2.1. Materials

Chitosan-oligosaccharide (COS, MW 5 kDa, degree of deacetylation = 90%) was obtained from Zhejiang Golden-Shell Pharmaceutical Co., Ltd. (Yuhuan, China). Thyme oil (TO) and polyvinyl alcohol (PVA, 1799) were purchased from Macklin Biochemical Co., Ltd. (Shanghai, China). Cinnamaldehyde (CA, purity ≥ 98%) was provided by Aladdin Reagent Co., Ltd. (Shanghai, China). Potato dextrose agar (PDA) and potato dextrose broth (PDB) were obtained from Bowei Biotechnology Co., Ltd. (Shanghai, China). *Penicillium italicum* (*P. italicum*) was isolated and purified by the Key Laboratory of Horticulture Science, Southwest University (Chongqing, China). Chemicals, other than those used in this study, were of analytical grade.

### 2.2. Preparation of the Thyme Oil Emulsion

The TO emulsion was prepared using a two-step emulsification method with some modifications [6,11]. The oil phase consisted of a mixture of TO and CA, where the concentration of TO was set to 1 wt%, 2 wt%, and 4 wt% in the emulsion (denoted as 1TO-E, 2TO-E, and 4TO-E), and the concentration of CA was 2.5 wt% of the oil phase. The aqueous phase was a mixed solution of COS and PVA at pH 6.5, with a COS concentration of 0.5 wt% and a PVA concentration of 1 wt%. The first step was to introduce the oil phase into the aqueous phase gradually, and the resulting mixture was homogenized using a high-speed disperser (FJ200-S; Lichen Instrument Technology Co., Ltd., Changsha, China) at a speed of 12,000 rpm for 3 min in an ice bath. Subsequently, the obtained coarse emulsion was subjected to ultrasonication using a 20 kHz ultrasonic processor (Scientz-II D, Ningbo Scientz Biotechnology Co., Ltd., Ningbo, China) at a power output of 300–600 W for 5, 10, and 20 min in an ice bath, with a 5 s ultrasound pulse followed by a 5 s pause. The pH was then adjusted to 4 with 0.1 M hydrochloric acid solution to obtain the final emulsion. The pH was adjusted from 6.5 to 4 to enhance COS protonation, promoting stronger electrostatic interactions at the oil–water interface and improving emulsion stability. For storage stability tests, emulsions were stored in sealed glass containers at 25 °C in the light-avoiding environment and sampled at 1, 7, and 21 d for analysis.

### 2.3. Physicochemical Characterization of Thyme Oil Emulsion

#### 2.3.1. Droplet Size and Zeta Potential Measurement

The droplet size, polydispersity index (PDI), and zeta potential of the emulsions were measured using a dynamic light scattering (DLS) instrument (Zetasizer Nano, Malvern Instruments Ltd., Malvern, Worcestershire, UK). To minimize the effects of multiple scattering, the original samples were diluted with deionized water before the measurement. All measurements were conducted at 25 °C and repeated three times.

#### 2.3.2. Microscopy Observation

To observe the morphology of an emulsion using an optical microscope (BX53, OLYMPUS, Tokyo, Japan), samples were prepared by placing 5–10 μL of emulsion on a clean glass slide, diluting if necessary, and covering it with a coverslip. Using a bright-field microscope, the observation started with a low magnification (10×) to locate the sample and gradually increased to higher magnifications (40× or 100×). A digital camera was used to capture the microscopic images of emulsions. At least three independent samples were prepared to ensure reproducibility.

#### 2.3.3. Encapsulation Efficiency Determination

The encapsulation efficiency (EE) of TO in the COS-stabilized emulsions was determined using a modified centrifugation method. Briefly, the freshly prepared emulsion (5 mL) was transferred into a centrifuge tube and centrifuged at 10,000 rpm for 30 min at 4 °C to separate the free (non-encapsulated) TO. The supernatant containing the free TO was carefully collected and extracted with an equal volume of ethanol under gentle agitation to ensure complete oil dissolution. The concentration of free TO was quantified spectrophotometrically at the characteristic absorption wavelength (274 nm), using a standard calibration curve. The EE of TO in the emulsion was calculated using the following formula:

$$Encapsulation\;efficiency\left(\%\right)=(\frac{C_{t}-C_{0}}{C_{t}})\times 100$$

where *C_t_* is the total concentration of TO in the emulsion and *C*_0_ is the concentration of free TO in the aqueous phase. All measurements were performed in triplicate.

### 2.4. Antioxidant Quenching Activity of Thyme Oil Emulsion

The antioxidant activities of emulsions were evaluated by ABTS^+^ and DPPH radical scavenging methods with minor modifications [17,18]. The emulsion sample (0.1 g) was diluted with 5 mL of deionized water. Subsequently, the diluted emulsion and ABTS/DPPH assay solution were mixed at a volume ratio of 1:1 and incubated for 60 min at room temperature in a dark environment. Finally, a UV-vis spectrophotometer (UV-2550, Shimadzu Inc., Kyoto, Japan) was used to measure the absorbance at 734 nm and 517 nm. The radical scavenging activity was calculated using the following equation:

(1) $$Radical\;scavenging\;activity\left(\%\right)=(1-\frac{A_{1}}{A_{2}})\times 100$$

where *A*_1_ is the absorbance of the solutions treated with TO/TO emulsions. *A*_2_ is the absorbance of control solutions (without TO/TO emulsions, i.e., only DPPH or ABTS solutions).

### 2.5. Antimicrobial Activity of Thyme Oil Emulsion

#### 2.5.1. Determination of Minimum Inhibitory Concentration (MIC) and Minimum Bactericidal Concentration (MBC)

The MIC and MBC of the emulsions were determined using a microdilution method with iodonitrotetrazolium chloride (INT) as a chromogen in sterile 96-well plates against *Escherichia coli* (*E. coli*), *Staphylococcus aureus* (*S. aureus*), and *Penicillium italicum* (*P. italicum*) with slight modifications [11,19]. Firstly, the samples were serially diluted two-fold in the wells. Then, 100 μL of bacterial or spore inoculum (10^4^–10^5^ CFU/mL) was added to each well, ensuring complete mixing to achieve final concentrations of TO emulsion between 10,000 μg/mL–78.125 μg/mL. For bacterial testing, the plates were incubated at 37 °C for 24 h, followed by adding 50 μL of 0.2 mg/mL iodonitrotetrazolium chloride (INT) chromogen. After 30 min incubation at 37 °C, the MIC was determined as the lowest concentration showing no color change or visible bacterial growth. The plates were incubated at 28 °C for 48 h for fungal testing, with fungal growth being observed. The MIC was defined as the lowest concentration without any precipitation. The non-discolored or non-precipitating wells were then transferred to fresh medium, with bacterial and fungal cultures incubated at 37 °C for 24 h and 28 °C for 48 h, respectively. The MBC was the lowest concentration at which no microbial growth was observed.

#### 2.5.2. Determination of the Antibacterial Inhibition Zone of the TO Emulsion

The antibacterial activity of the TO or TO emulsions was evaluated using the inhibition zone method with an Oxford cup against *E. coli* and *S. aureus*. Bacterial strains were cultured in Luria–Bertani (LB) broth at 37 °C overnight to a concentration of approximately 1 × 10^7^ CFU/mL. Sterile Mueller–Hinton agar (MHA) plates were prepared, and 100 µL of the bacterial suspension was evenly spread across the surface. After the agar was surface dried for 5 min, sterile Oxford cups were placed on the agar, and each cup was filled with 100 µL of the test samples. The plates were incubated at 37 °C for 24 h under aerobic conditions. Zones of inhibition around the Oxford cups were measured in millimeters using a digital caliper, with measurements averaged from three replicates.

#### 2.5.3. Inhibitory Effect of the Emulsion on Mycelial Growth of *P. italicum*

The inhibitory effect of the TO emulsion on the mycelial growth of *P. italicum* was assessed following a previous method with minor modifications [20]. Briefly, sterilized and cooled potato dextrose agar (PDA) medium was thoroughly mixed with the free TO or TO emulsion and poured into several Petri dishes. Plates containing only PDA medium served as controls. Once the medium had solidified, a 7-day-old mycelial disk (5 mm in diameter) of *P. italicum* was placed at the center of each dish. All plates were incubated at 28 °C for one week. Colony diameters were measured and recorded after 3 d of incubation. The inhibition rate of mycelial growth was calculated using the following formula:

(2) $$Inhibition\;rate\;of\;mycelial\;growth\left(\%\right)=\frac{d_{1\;}-{\;d}_{2}}{d_{1}\;-\;d_{0}}\times 100$$

where *d*_1_ and *d*_2_ represent the average colony diameters of the control and treated groups, respectively, while *d*_0_ denotes the initial diameter of the mycelial disk, respectively.

### 2.6. Statistical Analysis

All experiments were conducted in triplicate, and the results are presented as mean ± standard deviation. Statistical analyses were performed using SPSS software (version 25.0, IBM, Armonk, NY, USA). Data were evaluated using analysis of variance (ANOVA), with statistical significance defined at *p* < 0.05.

## 3. Results and Discussion

### 3.1. Formation of Thyme Oil-in-Water Emulsions

A COS-stabilized TO-only oil phase emulsion was prepared using a two-step emulsification method, and the droplet sizes were effectively controlled using the ultrasound-assisted method. At lower ultrasound power (300 W), the size of TO-in-water emulsion was larger, with a droplet size range of 520.8 to 719.08 nm (Table 1). With the increase in ultrasonic power, the droplet size of the emulsion decreased significantly to less than 250 nm, which might be due to the exposure of more groups in ultrasonic-mediated unfolding and depolymerization of the COS chain, so it has more opportunities to react with CA to form a stable interfacial layer [15,21,22]. As ultrasound power increased, the number of generated cavitation bubbles also increased, resulting in enhanced energy around the bubbles, facilitating droplet dispersion, reducing droplet size, and improving emulsion stability [23,24]. This result is consistent with previous research reports, indicating that higher ultrasonic power dispersed oil droplets and helped to form emulsions with smaller droplet sizes [25,26]. Notably, although increasing the ultrasound power further resulted in a slight reduction in the droplet size of the emulsion, this change was deemed unnecessary from an energy-saving perspective [23]. Additionally, the ultrasonic treatment time also significantly impacted the size of emulsion droplets, because the droplet size decreased significantly from 404.75 nm to 224.27 nm as the treatment time increased from 5 min to 10 min. The cavitation effect of ultrasound facilitated the uniform dispersion of the water phase and the fragmentation of the oil phase, which increased the oil/water interface area and enhanced the interaction between the phases. As a result, COS rapidly accumulated with CA at the oil–water interface, which may form a solid-like interface layer similar to chitosan, exerting its emulsifying effect with PVA [11]. This method of using co-emulsifiers (COS/CA and PVA) in the interfacial phase is a very effective way to prevent Ostwald ripening [11,27]. As the processing time increased from 10 min to 20 min, there was no significant change in droplet size. Additionally, the lowest PDI values were observed at 10 min of processing under higher power conditions, indicating that this combination achieved the most uniform droplet size distribution (Table S1). Although the mean droplet size showed no significant difference between 10 and 20 min at higher powers, the slight increase in PDI at 20 min suggested the onset of destabilization, which might be due to the aggregation of COS and droplet coalescence, ultimately compromising the stability of the emulsion [25,26]. The encapsulation efficiency of TO in the emulsions was assessed, achieving approximately 94.74% at a 1% TO level and 91.73% at a 2% level (Figure S1), indicating the high payload and potential protection of TO. Finally, the optimized conditions (450 W, 10 min) were selected for all subsequent analyses based on achieving the smallest droplet size with a narrow size distribution and good stability.

### 3.2. Storage Stability of Thyme Oil-in-Water Emulsions

#### 3.2.1. Appearance and Droplet Size Change of TO Emulsions

**Figure 1:**
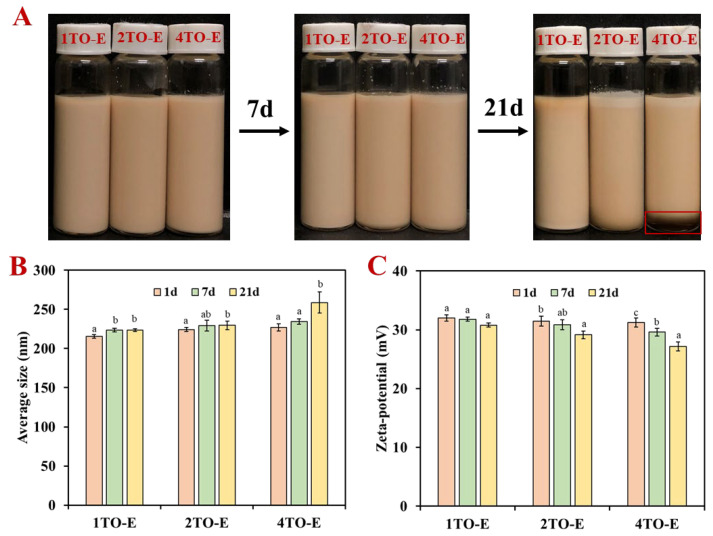
(**A**) Appearance stability, (**B**) droplet size, and (**C**) zeta potential of TO-in-water emulsions under different TO concentrations. Different letters within the same TO concentration indicate statistically significant differences (*p* < 0.05). 1TO-E, 2TO-E, and 4TO-E refer to 1%, 2%, and 4% (wt) of TO in the emulsions, respectively.

The appearance of TO-in-water emulsions was uniform and light brown, and there was no change in all TO emulsions within 7 d, which indicated that TO emulsions remained visually stable across varying TO concentrations (Figure 1A). However, the emulsion with high TO concentration (4TO-E) appeared to undergo slight creaming and phase separation at 21 d (red frame), and the droplets floated without demulsification and oil leakage, which was related to the lower density of TO and the influence of gravity on the oil droplets [28,29] This result also indicated that the stability of high-TO emulsions had relatively reduced. Notably, the absence of phase separation suggested effective stabilization by the COS/CA solid-like interface layer and PVA, and the presence of PVA also improved the viscosity of the emulsion and weakened the separation effect of gravity [11,30].

The droplet size analysis, based on DLS measurements, showed a correlation between TO concentration and emulsion droplet size (Figure 1B). The initial droplet size did not differ significantly among TO concentrations, although a slight increasing trend was observed with higher oil phase content, where the average droplet size was approximately 215 nm at a TO concentration of 1%. Increasing the TO concentration to 2% and 4% slightly increased the droplet size to about 225 nm, likely due to the increased viscosity of the dispersed phase (Figure S2). A higher viscosity in the continuous phase can slow down droplet movement and reduce gravitational separation (creaming), thereby enhancing emulsion stability. This relationship between viscosity and droplet mobility is consistent with previous findings in essential oil-in-water emulsion stability studies [11]. The droplet size of TO-in-water emulsions had no noticeable change within one week and remained within 240 nm (Figure 1B). During the longer-term storage for 21 d, the droplet size of 1TO-E and 2TO-E emulsions remained stable and did not increase significantly, while that of 4TO-E emulsions increased to 258.66 nm. These minor changes might suggest minimal coalescence or Ostwald ripening, reflecting the effectiveness of the COS/CA solid-like interfacial layer and PVA stabilization mechanism [11,15]. Despite this increase, the droplet sizes remained within a range indicative of good emulsion stability. Zeta potential values revealed that the emulsions maintained high absolute values, exceeding 30 mV across all TO concentrations within 7 d of storage (Figure 1C). The consistent zeta potential across TO concentrations indicated that the COS/CA solid-like interface layer and PVA effectively stabilized the system regardless of oil loading [31]. After 21 days of storage and at a TO concentration of 1% and 2%, the zeta potential was around 29.13–32 mV (Figure 1C), ensuring strong electrostatic repulsion between droplets, which prevented aggregation and contributed to longer-term stability [27,32]. It should be noted that the zeta potential of 4TO-E emulsion decreased to 27.16 mV at 21 d, indicating the direct electrostatic repulsion of its droplets, which corresponds to the increase in its average size above. This phenomenon was similar to previous studies on sodium caseinate (self-assembled) and chitosan-stabilized TO emulsion [8,33].

#### 3.2.2. Distribution of Droplets During Storage of TO Emulsions

**Figure 2:**
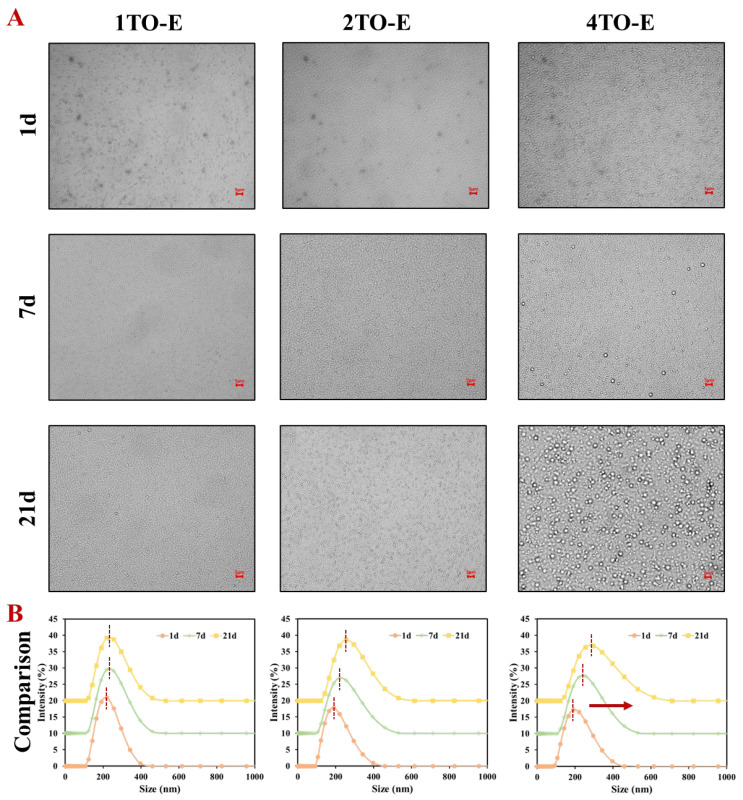
(**A**) Microscopic images and (**B**) droplet size distribution of TO-in-water emulsions during 21-day storage under different TO concentrations. The scale bars were 5 μm. The red arrow indicates the rightward shift of the peak position. 1TO-E, 2TO-E, and 4TO-E refer to 1%, 2%, and 4% (wt) of TO in the emulsions, respectively.

The droplet size distribution over a 21-day storage period highlighted the emulsion’s stability under different TO concentrations. All emulsions showed a single peak distribution within 21 d, corresponding to their uniform droplet size (Figure 2A). However, the peak position of the 4TO-E emulsion showed the most obvious shift to the right with increasing storage time (Figure 2B), indicating the droplet size increase, which was also observed in the previous reports on chitosan-stabilized essential oil emulsions [11,27]. The droplets of emulsions presented a uniform spherical shape. The morphology of the 1TO-E and 2TO-E emulsions had no obvious change in 21 d, while the droplets of the 4TO-E emulsion appeared larger from 7 d to 21 d, indicating that coalescence and Ostwald ripening occurred [34,35]. Collectively, the stability mechanism of TO emulsions can be divided into the following two aspects: (i) The combination of the COS/CA solid-like interface layer and PVA as co-stabilizers can effectively adsorb at the oil–water interface to improve the stability of TO-in-water emulsions; (ii) PVA can be used as a thickener to increase the viscosity of the continuous phase, weaken the influence of gravity on the emulsion droplet, and enhance its stability. Meanwhile, TO concentration had a pronounced effect on droplet size, stability, and functional performance, underscoring the need to optimize oil content for targeted application requirements. Therefore, comparisons of functional properties were made between free TO (2%) and encapsulated emulsions at 1% and 2% TO, which exhibited stable physicochemical properties. In addition, ultrasonication significantly contributed to reducing droplet size and improving emulsion homogeneity. The ability of TO emulsions to maintain droplet size and distribution over time highlights their potential practical commercial use.

### 3.3. Antioxidant Activity of Thyme Oil-in-Water Emulsions

**Figure 3:**
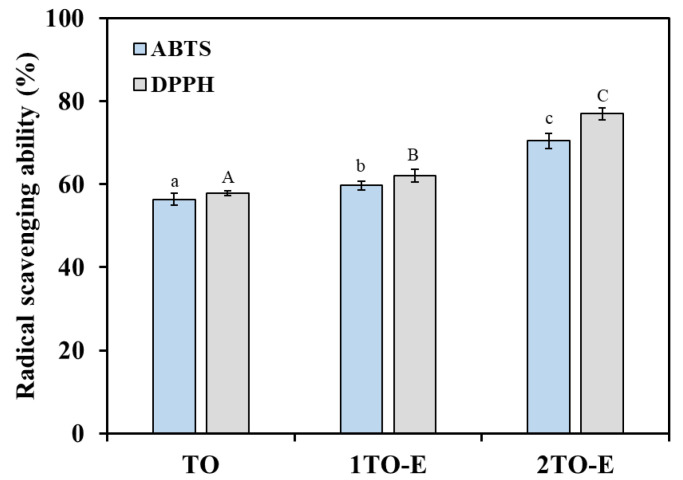
Antioxidant activity of free and encapsulated TO under different concentrations. TO refers to 2% (wt) of TO in ethanol solution, and 1TO-E and 2TO-E refer to 1% and 2% (wt) of TO in the emulsions, respectively. Lowercase letters represent significant differences in ABTS assay results, whereas uppercase letters correspond to differences in DPPH assay (*p* < 0.05).

The antioxidant activity of free and encapsulated TO was evaluated by ABTS and DPPH radical scavenging activity, which is depicted in Figure 3. Free TO had good antioxidant capacity, with its ability to scavenge ABTS and DPPH free radicals reaching 56.3% and 57.79%, respectively, at a concentration of 2%. Noticeably, encapsulation significantly enhanced the antioxidant potential compared to free TO across all tested concentrations. At a concentration of 1%, the scavenging activity of encapsulated TO against DPPH free radicals was approximately 62.1%, slightly higher than the scavenging activity of free TO at a concentration of 2%. Meanwhile, at a concentration of 2%, the ABTS and DPPH radical scavenging activities of encapsulated TO reached approximately 70.45% and 76.92%, respectively, which were 1.25- and 1.33-fold higher than the free TO at the same concentration, respectively. This improvement was likely due to the protective effect of the COS/CA solid-like interface layer and PVA matrix, which minimized oxidation and preserved the functional components of TO. Numerous studies have shown that encapsulation by natural polymer emulsions enhanced the scavenging abilities of essential oils, aligning with the findings of this research [18,36,37]. Interestingly, the emulsions demonstrated a more substantial free radical scavenging capacity against DPPH compared to ABTS, which could be attributed to the alcohol solubility of TO [17,38]. Generally, the DPPH radical scavenging activity of essential oils is often higher than that of ABTS due to the better solubility of lipophilic components in the organic solvents used in the DPPH assay. Moreover, the hydrogen atom transfer (HAT) mechanism of DPPH better matches the antioxidant behavior of terpenoids commonly found in essential oils. Browning and lipid oxidation are significant challenges in fresh food storage, with free radical scavenging being a key mechanism through which antioxidants inhibit lipid oxidation [39]. Therefore, due to its strong antioxidant capacity, using TO emulsion as a coating or packaging material for fresh food offers an effective strategy to slow down the quality deterioration during storage.

### 3.4. Antimicrobial Activity of Thyme Oil-in-Water Emulsions

#### 3.4.1. MIC and MBC Evaluation of TO Emulsions

MIC and MBC serve as key indicators for evaluating the antimicrobial activity of essential oils. MIC refers to the minimum concentration required to inhibit pathogenic microorganisms’ growth or halt their survival, while MBC is the minimum concentration needed to eliminate 99.9% of pathogenic microorganisms. Lower MIC and MBC values indicate a more substantial antimicrobial effect [11]. TO can effectively suppress the growth and proliferation of bacteria and molds by causing cell membrane invagination, organelle disintegration, and compromising the integrity of cell structures [40]. For all tested strains, the MIC values for the TO emulsions (312.5 μg/mL) were half of the MIC value for free TO (625.0 μg/mL) at a 2% concentration, indicating that the emulsion formulation enhanced the antimicrobial efficiency by requiring lower concentrations to inhibit microbial growth (Table 2). Similarly, the MBC values demonstrated that the TO emulsion was more effective in achieving complete microbial eradication than free TO.

The enhanced antimicrobial activity of the COS emulsion systems could come from three primary mechanisms. Firstly, the improved performance could be attributed to the enhanced solubility and stability of TO within the emulsion system, which facilitated better interaction with microbial cells and more effective delivery of the active compounds [41]. Furthermore, the encapsulation likely enabled a sustained release of TO, maintaining an active concentration over time and preventing rapid evaporation or degradation [42]. Secondly, the positively charged emulsion droplets adhered to the negatively charged surfaces of microorganisms, promoting interactions with surface proteins. This interaction increased membrane permeability and disrupted cell wall integrity, thereby amplifying the antimicrobial effects of TO [18,43]. Thirdly, the reduction in emulsion droplet size and the increase in specific surface area, achieved via ultrasonic-assisted homogenization, improved the penetration efficiency of antimicrobial components into the cell membrane, which enhanced their overall effectiveness [37,44]. These results were consistent with previous studies that demonstrated the positive impact of emulsification on the antimicrobial activity of TO. Similar to the findings from a study on double-layer TO emulsions stabilized by whey protein and chitosan, which enhanced both stability and antimicrobial efficacy, an increase in antimicrobial activity was observed [31]. However, in contrast to this method, significant antimicrobial effects were achieved using a single-layer emulsion without ripening inhibitors. This approach offers an advantage over more complex formulations, as ripening inhibitors, such as corn oil or medium-chain long triglycerides, have been shown to reduce the antimicrobial efficacy of TO emulsions, as reported in another study [1]. Therefore, the simpler formulation not only maintains antimicrobial activity but also avoids the potential negative impact of complex structural designs or stabilizers on bioactivity.

#### 3.4.2. Inhibition Zone Evaluation of TO Emulsions

**Figure 4:**
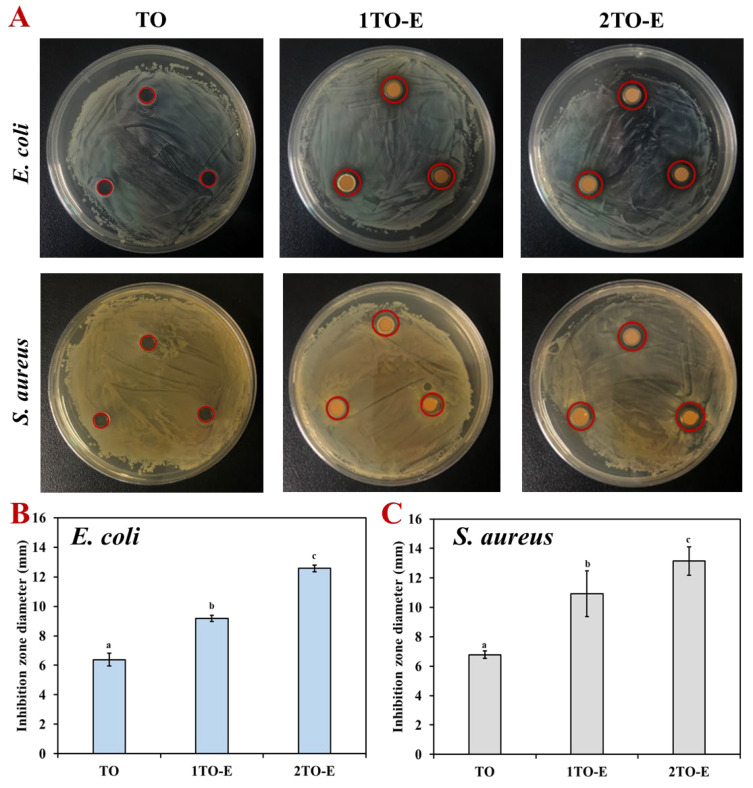
Inhibition zone appearance (**A**) and diameters (**B**,**C**) of free and encapsulated TO against *E. coli* and *S. aureus* under different concentrations. The red circles highlight the diameter of the inhibition zones. Different letters within the same test indicate statistically significant differences (*p* < 0.05).

The antibacterial ability of TO was evaluated in both free and emulsion-encapsulated forms against *E. coli* (Gram-negative) and *S. aureus* (Gram-positive) using the inhibition zone method. As shown in Figure 4, free TO at a concentration of 2% has a particular antibacterial effect, with inhibition zones of 6.38 mm and 6.77 mm against *E. coli* and *S. aureus*, respectively. Additionally, the inhibition zone diameters varied depending on the concentration of TO emulsions. For *E. coli*, 1TO-E and 2TO-E were 9.18 and 12.58 mm, respectively, and for *S. aureus*, the diameters were 10.94 and 13.15 mm, respectively. Generally, larger inhibition zones were observed with higher TO concentrations, indicating a dose-dependent antibacterial effect [45].

Regardless of the concentration, the encapsulated TO consistently exhibited larger inhibition zones than free TO. For example, at the same concentration of 2% TO, the inhibition diameters of emulsions were 1.97- and 1.94-fold larger than those of free TO, indicating a superior antibacterial effect (Figure 4B). This enhanced efficacy of encapsulated TO can be attributed to the emulsion system’s ability to improve the solubility and stability of TO, thereby facilitating better interaction with bacterial cells [8,33]. In addition, encapsulation likely aids in the controlled release of TO, maintaining a sustained antimicrobial action, and preventing rapid volatilization or degradation of active compounds. In contrast, free TO may lose potency due to its hydrophobic nature and higher susceptibility to environmental factors [28,31]. Interestingly, *S. aureus* exhibited larger inhibition zones than *E. coli* at corresponding concentrations, suggesting that Gram-positive bacteria might be more susceptible to TO emulsions. This difference in susceptibility could be attributed to the structural differences in bacterial cell walls: the thicker peptidoglycan layer of Gram-positive bacteria might allow more interactions with the active compounds in the TO emulsions [5,6,17]. These findings emphasized the critical role of emulsions in enhancing the antibacterial activity of TO, making them a promising delivery system for essential oil-based antimicrobial agents.

#### 3.4.3. Inhibitory Effect of TO Emulsions on Mycelial Growth

**Figure 5:**
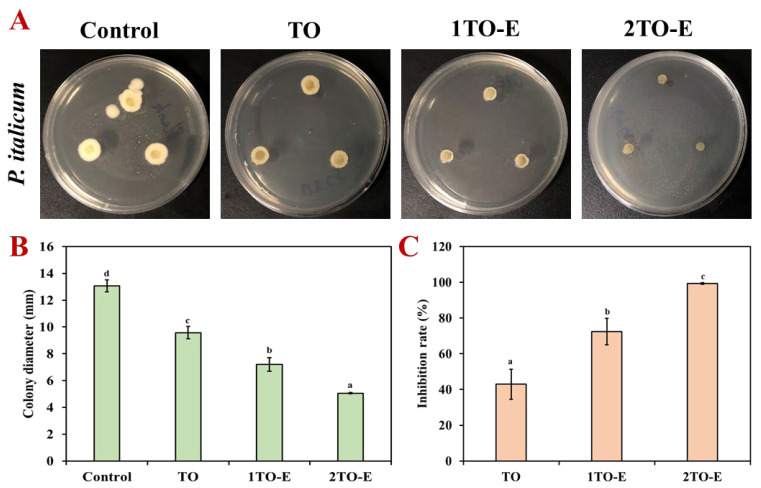
Colony appearances (**A**), inhibition diameters (**B**), and inhibition rates (**C**) of free and encapsulated TO against *P. italicum* under different concentrations. Different letters within the same test indicate statistically significant differences (*p* < 0.05).

The effects of free TO and TO emulsions on the growth of *P. italicum* mycelium were assessed by measuring the colony diameters under different concentrations, as shown in Figure 5. Both free and encapsulated TO exhibited inhibitory effects against *P. italicum* growth, with the inhibition increasing at higher concentrations (Figure 5A). The colony diameters of the 1TO-E and 2TO-E emulsions were 7.21 mm and 5.06 mm, respectively. Additionally, the TO emulsion demonstrated a significantly greater reduction in mycelium diameter compared to free TO across the same tested concentration (2%), where the colony diameter of TO was 9.58 mm, while that of the 2TO-E emulsion was 5.06 mm (Figure 5B).

Meanwhile, the inhibition rate of free TO was only 42.92%, while the antifungal effect was significantly improved after emulsion encapsulation (Figure 5C). The inhibition rate of 1TO-E increased to 72.4%, while 2TO-E reached 99.31%, which almost completely inhibited the growth of *P. italicum*. This enhanced antifungal activity could be attributed to TO’s improved dispersion and stability within the emulsion system, which allowed for better penetration and sustained interaction with fungal cells [4,20]. The encapsulated TO likely disrupted the fungal cell membrane more effectively, potentially due to the uniform release of active compounds over time. In contrast, free TO’s hydrophobic nature and volatility might limit its availability and efficacy [6,40]. These results highlighted the superior performance of TO emulsions in controlling fungal growth, suggesting their potential application as an effective antifungal treatment in agricultural practices or food preservation.

## 4. Conclusions

This study demonstrated the efficacy of COS-stabilized TO-in-water emulsions as a novel delivery system for enhancing the stability, antioxidant capacity, and antimicrobial activity of TO. Ultrasonic-assisted emulsification achieved fine droplet sizes and high encapsulation efficiencies, while the COS/CA interface layer and PVA contributed to longer-term stability and resistance to coalescence and Ostwald ripening. Emulsion encapsulation markedly improved the antioxidant and antimicrobial properties of TO, as evidenced by enhanced free radical scavenging activities and more substantial antibacterial and antifungal effects compared to free TO. The emulsions’ ability to inhibit microbial and fungal growth, coupled with their stable physicochemical properties, underscores their potential as an effective solution for applications in food preservation. Future work could explore the combination of these emulsions with coatings or other active packaging, as well as their scalability and practical deployment in commercial settings.

## Figures and Tables

**Table 1:** The droplet size (nm) of emulsions prepared using different ultrasonic power levels and processing time.

Time	Power		
	300 W	450 W	600 W
5 min	719.08 ± 4.87 ^f^	404.75 ± 3.11 ^c^	385.69 ± 4.42 ^b^
10 min	590.56 ± 4.33 ^e^	224.27 ± 2.72 ^a^	220.74 ± 3.89 ^a^
20 min	520.80 ± 2.29 ^d^	226.25 ± 6.01 ^a^	222.37 ± 2.86 ^a^

Notes: different letters of superscripts within the same test indicate statistically significant differences (*p* < 0.05).

**Table 2:** Minimum inhibitory concentration (MIC) and minimum bactericidal concentration (MBC) of free and emulsion (E)-encapsulated TO against *E. coli*, *S. aureus*, and *P. italicum*.

Microbial Strain	Antimicrobial Component	MIC (μg/mL)	MBC (μg/mL)
*E. coli*	2%TO in ethanol	625.0	1250.0
	2TO-E	312.5	312.5
*S. aureus*	2%TO in ethanol	625.0	625.0
	2TO-E	312.5	312.5
*P. italicum*	2%TO in ethanol	625.0	625.0
	2TO-E	312.5	312.5

## Data Availability

The data presented in this study are available on request from the corresponding author. (Please specify the reason for restriction, e.g., the data are not publicly available due to privacy or ethical restrictions).

## Supplementary Materials

The following supporting information can be downloaded at: https://www.mdpi.com/article/10.3390/foods14172930/s1, Table S1: The polydispersity index (PDI) of emulsions prepared using different ultrasonic power and processing time; Figure S1: Encapsulation efficiency of thymol-in-water emulsions under different TO concentrations; Figure S2: Viscosity of thymol-in-water emulsions under different TO concentrations. Different letters within the same TO concentration indicate statistically significant differences (*p* < 0.05).
