# Supplementary material for: Ultrasound-Assisted Preparation of Chitosan Oligosaccharide-Stabilized Thyme Oil-in-Water Nanoemulsions: Enhanced Storage Stability and Antimicrobial Properties
Source: Foods. 2025 Aug 22;14(17):2930. doi: 10.3390/foods14172930 (PMC12428331; doi:10.3390/foods14172930)

Supplementary Materials

Supplementary Methods

1. Viscosity measurement of TO emulsions

The apparent viscosity of the TO emulsions was measured at 25 °C using a rotational viscometer (NDJ-8S, Shanghai Precision Instrument Co., Shanghai, China) equipped with the appropriate spindle for low-viscosity liquids. Each freshly prepared emulsion sample (1TO-E and 2TO-E) was gently mixed to ensure homogeneity before measurement. The viscosity was recorded at a constant rotational speed of 60 rpm, and results were expressed in mPa·s. All measurements were performed in triplicate, and the average values were used for analysis.

Supplementary Table and Figures

Table S1. The polydispersity index (PDI) of emulsions prepared using different ultrasonic power and processing time.

Figure S1. Encapsulation efficiency of thymol-in-water emulsions under different TO concentrations.

Figure S2. Viscosity of thymol-in-water emulsions under different TO concentrations. Different letters within the same TO concentration indicate statistically significant differences ($P < 0.05$).

**Time**	**Power**		
	300 W	450 W	600 W
5 min	0.369±0.05	0.193±0.013	0.186±0.03
10 min	0.311±0.023	0.084±0.015	0.089±0.021
20 min	0.252±0.021	0.121±0.023	0.103±0.01

Figure S1.

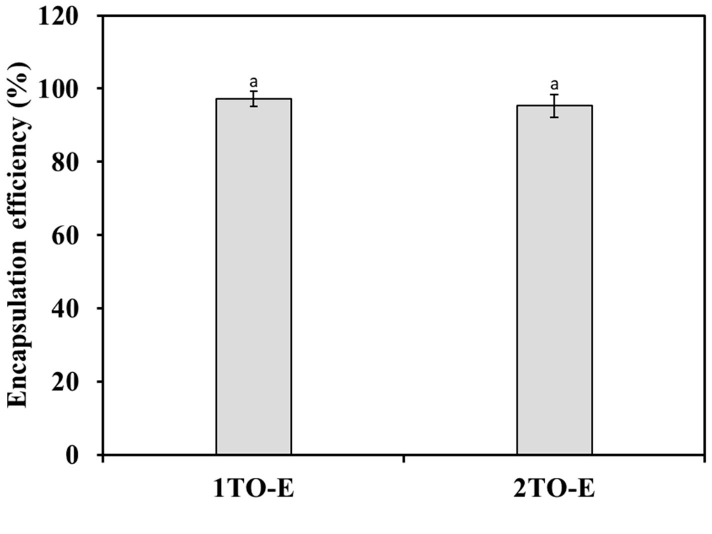

Figure S2.

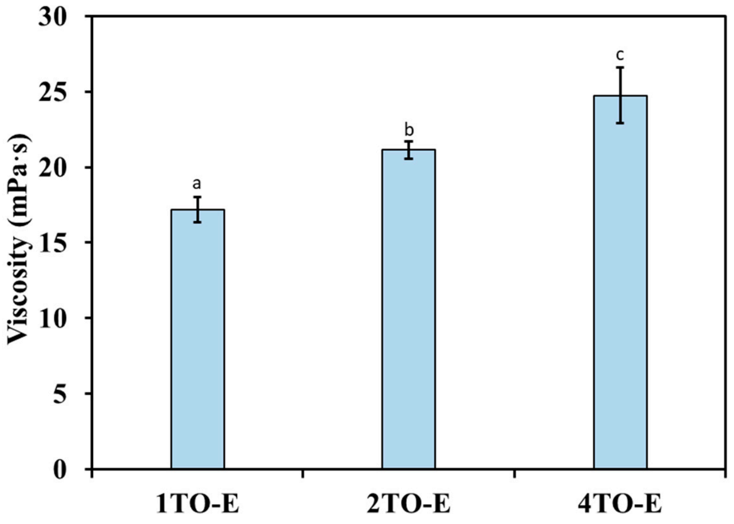

Supplement: Supplementary file 1 [file foods-14-02930-s001.zip › foods-3813994-supplementary.pdf]
